# Improving Early Initiation of Breastfeeding in Southeast Asia: The Alive & Thrive Experience

**DOI:** 10.1089/bfm.2018.0125

**Published:** 2018-10-18

**Authors:** Kristina Granger

**Affiliations:** Alive & Thrive, FHI 360, Washington, District of Columbia.

**Keywords:** EIBF, EENC, newborn, C-section, Southeast Asia

In 2013, the rate of neonatal deaths in the Western Pacific Region was 230,000 per year.^1^ Every 2 minutes, a newborn died. Two out of three of those deaths occurred in the first 3 days of life, many from preventable causes.^1^ Evidence showed that delaying the initiation of breastfeeding by >1 hour in the first day of life could double the risk of dying from infection-related deaths, whereas delaying between 24 and 48 hours almost tripled the risk.^2^ The prevalence of early initiation of breastfeeding (EIBF), within the first hour of life, varied widely in the region, with high rates in Samoa (88%) and the Solomon Islands (75%), and a much lower rate in Laos (30%).^3^ Babies who were not exclusively breastfed in the first month had four times the risk of infection-related death as those who were exclusively breastfed.^4^ Exclusive breastfeeding rates, at the time, also varied with the Philippines (34%), Laos (26%), and Viet Nam (17%) having the lowest rates in the region.^3^

Alive & Thrive (A&T) began working to improve breastfeeding in Viet Nam in 2009, when the annual rate of newborn death was ∼17,500 newborns.^5^ Working closely with the government and other partners, A&T implemented a social franchise model for improving infant and young child feeding counseling, a mass media campaign, and a series of policy and advocacy initiatives to support an enabling environment for breastfeeding. An impact evaluation of the program found that rates of exclusive breastfeeding had increased from 18.9% to 57.8% in A&T-supported program areas.^6^ Staff at health facilities participating in the program demonstrated significantly better infant feeding knowledge and interpersonal communication skills than staff in nonprogram facilities. However, despite improvements in exclusive breastfeeding rates, data showed that EIBF did not improve. In Viet Nam, although 93.8% of births have a skilled attendant at delivery, only 26.5% of infants began breastfeeding within 1 hour of birth.^7^ Historically, it had been common practice in facilities to separate the baby from the mother to perform various tasks before breastfeeding was initiated. Although the A&T program helped mothers and caregivers understand the importance of exclusive breastfeeding and how to improve practices at home, it did not specifically address provider practices related to initiating breastfeeding within 1 hour of birth.

## Early Essential Newborn Care

In 2014, awareness was increasing of the importance of standardizing and strengthening the care provided to newborns at birth. World Health Organization (WHO) and the United Nations Children's Fund (UNICEF) finalized the *Action Plan for Healthy Newborn Infants in the Western Pacific Region (2014–2020)*, which outlined a set of key practices for early essential newborn care (EENC). The *Action Plan* included guidelines and targets for EENC for all member states in the region, as well as care guidelines for preterm, low-birth weight infants, and sick newborns. The EENC intervention package includes immediate and thorough drying of the newborn, immediate skin-to-skin contact, delayed cutting of the umbilical cord until pulsations stops, and EIBF.^1^ WHO's “The First Embrace” initiative promoting immediate skin-to-skin contact soon after the baby is born, and emphasizing nonseparation of mother and baby until after completion of the first breastfeed, also started during this period.

## Improving Newborn Health in Viet Nam

With the support from the WHO Western Pacific Region, the Viet Nam Ministry of Health (MoH) adapted the regional *Action Plan* for EENC and piloted EENC guidelines specific to Viet Nam, focusing on the practice of EIBF for vaginal deliveries in 2014, and cesarean (C-section) deliveries in 2016.^8^ Along with UNICEF and WHO, A&T supported the MoH and provincial departments of health in capacity building and rolling out the EENC guidelines in facilities across almost two-thirds of Viet Nam's 63 provinces and cities. A&T supported local departments of health and reproductive health centers in seven provinces by using national trainers and local champions to train facility staff in the new guidelines, and put in place coaching and supportive supervision mechanisms at the facilities. In total, A&T supported training, coaching, and supportive supervision for 779 health staff in 102 hospitals as part of the roll out of the EENC guidelines for vaginal deliveries. Across all provinces in the health facilities supported by A&T, EIBF rates improved significantly after the capacity-building activities and continued to remain high. EIBF for vaginal deliveries reached 90% or higher in the supported health facilities. Despite improvements in EIBF postvaginal birth, data showed a significant gap in EIBF for C-section deliveries (only 30%). Monitoring data revealed barriers to following EENC guidelines during post-C-section, including limited provider skills and a lack of sufficient personnel to facilitate EENC during C-sections. To help improve this practice, utilizing its network of national trainers, A&T supported capacity building for implementing the national guidelines of EENC for C-section deliveries in five provinces, reaching 66 hospitals and 593 health staff. These efforts produced significant improvements in EIBF, closing the gap between vaginal and C-section deliveries.

## Lessons for Success

Several factors enabled the widespread change in provider behavior that resulted from the effective implementation of the EENC guidelines in Viet Nam. Having explicit national-level EENC policies for both vaginal and C-section deliveries increased provider adherence to key EENC actions at delivery. In addition, securing support from a dedicated group of breastfeeding champions in both the government and the facilities helped to get the policies implemented. These champions, along with active leadership across facilities, held practitioners accountable to the new policies. The on-site coaching and supportive supervision provided opportunities for regular feedback to continually strengthen health worker capacity. Regular monitoring data allowed facility staff and the program team to reward high-performing facilities, and redirect training and support resources to those institutions that were performing poorly. Other efforts were simultaneously working to improve the enabling environment and demand for EENC, for instance the regional “First Embrace” initiative, as well as national-level interpersonal communication and mass media activities promoting EENC and EIBF.

The development and rollout of EENC guidelines and policies have been a critical component of broader efforts to improve breastfeeding and impact child health outcomes in Southeast Asia. Evidence of the protective effect of early breastfeeding on newborns is clear, and data indicate that EIBF is a strong predictor of successful exclusive breastfeeding to 6 months. With the high prevalence of facility-based deliveries and growing rates of C-section deliveries in the region, institutionalizing EIBF through EENC is an effective way to ensure that babies survive and thrive.

## Scaling Up in the Region

A&T is continuing to work with UNICEF, WHO, and stakeholders in the region to improve maternal, infant, and young child nutrition. This strong network of partnerships in the region aims to create an enabling environment for improved breastfeeding through stronger legislation, increased resource allocation, and more accountability to nutrition outcomes. A&T directly supports health systems strengthening to deliver high-quality breastfeeding-friendly services to mothers and infants in Cambodia, Laos, Myanmar, and Viet Nam, including providing technical assistance to establish and sustain a network of 30–40 Centers of Breastfeeding Excellence as model EENC service providers. A&T also works with partners to advocate for national laws and policies around maternity protections and for stronger regulations on the marketing of breast milk substitutes. These efforts contribute to the remarkable progress being made in policies and programs that protect breastfeeding.
